# Thinking in action: Need for Cognition predicts Self-Control together with Action Orientation

**DOI:** 10.1371/journal.pone.0220282

**Published:** 2019-08-01

**Authors:** Julia Grass, Florian Krieger, Philipp Paulus, Samuel Greiff, Anja Strobel, Alexander Strobel

**Affiliations:** 1 Department of Psychology, Chemnitz University of Technology, Chemnitz, Germany; 2 Cognitive Science and Assessment, University of Luxembourg, Esch-sur-Alzette, Luxembourg; 3 Faculty of Psychology, Technische Universität Dresden, Dresden, Germany; University of Lleida, SPAIN

## Abstract

Need for Cognition describes relatively stable interindividual differences in cognitive motivation. Previous research has shown relations of Need for Cognition to Self-Control–a capacity that can be broadly defined as resistance to temptation–yet, the processes underlying this relation remain unclear. One explanation for the prediction of Self-Control by Need for Cognition can be an increased motivation to invest cognitive effort with higher levels of Need for Cognition. Another possible link could be that individual differences in the implementation of Self-Control intentions may play a moderating or mediating role for the predictive value of Need for Cognition. Such individual differences in the self-motivated initiation and maintenance of intentions are described by dispositional Action Orientation. Therefore, in the present study, Action Orientation was examined with regard to its possible role in explaining the relation of Need for Cognition to Self-Control. In a sample of 1209 young adults, Self-Control was assessed with two different self-report instruments and moderation and mediation models of the relationship between Need for Cognition, Action Orientation, and Self-Control were tested. While there was no evidence for a moderating role of Action Orientation in explaining the relation of Need for Cognition and Self-Control, Action Orientation was found to partly mediate this relation with a remaining direct effect of Need for Cognition on Self-Control. These results add to the conceptual understanding of Need for Cognition and demonstrate the relevance of trait variables to predict Self-Control.

## Introduction

Imagine, there is a delicious marshmallow right in front of you. The experimenter has gone out of the room and just told you that you will get two of the tasty marshmallows if you do not eat the one next to you until he returns. Otherwise, you can ring a bell for his immediate return. In this case, you will get no additional marshmallow. Of course, you would like to have two marshmallows instead of one, but will you be able to wait in face of the deliciously smelling one right in front of you?

This dilemma between getting one reward now versus a larger one later illustrates a basic characteristic of tasks with demands on Self-Control: the conflict between long-term global goals or values and short-term situational benefits [1]. Besides situational influences, people differ in their ability to withstand impulses and to resist temptations (e.g., [2]). To explain such differences, different theoretical models have been proposed. In general, all these approaches address that to control impulses is effortful and that subjective costs of Self-Control explain why people fail or succeed in Self-Control (e.g., [3,4]). Research concerning personality variables could show that individuals differ in the value they are attributing to cognitive effort and that they tend to invest cognitive resources differently [5]. Focusing on a personality perspective, such dispositional differences are described by so-called investment traits that “determine when, where, and how people invest their time and effort in their intellect” ([5], p. 841).

One well-examined investment trait that has been already linked to self-control is *Need for Cognition (NFC*). NFC was introduced as “differences among individuals in their tendency to engage in and enjoy thinking” ([6], p. 116). Since then, NFC has been examined in a large number of studies, especially focusing on its relations to individual differences in information processing [7] as well as to other cognitive (e.g., [8,9]) and personality variables [10,11]. In general, NFC describes the degree to which intrinsic value is assigned to cognitive activity as well as the amount of effort typically invested in cognitive endeavors [6]. Individuals with higher NFC levels approach cognitively challenging tasks and process information in a rather elaborated way [7]. In comparison, lower levels of NFC are associated with a rather heuristic information processing style and with the avoidance of cognitive effort [7]. Referring to personality, NFC is positively associated with Openness to Ideas, with Emotional Stability, and with traits indicating goal-oriented behavior [10]. It is related to academic achievement and decisional processes [5,7,12], but clearly distinguishable from intelligence [5,9,10,13]. There is also some evidence linking NFC to desirable non-cognitive outcomes like positive emotionality, affective adjustment, and self-esteem (e.g., [10,14,15]). For example, individuals with higher NFC levels report to be more satisfied with their studies [16] and with their life in general [17]. The results of two studies point to an increased recruitment of resources when cognitive demands are high: Referring to neural activity, a recent EEG study (*N* = 42) reported that individuals with higher NFC levels responded to increased cognitive demands with recruiting relatively more cognitive resources whereas individuals with low NFC levels did not show a comparable pattern of cognitive activity [18]. Another experimental study (*N* = 46) that examined how NFC relates to Self-Control showed a positive association between NFC and the performance in a Stroop test measuring the inhibition of predominant responses after they had to solve another mentally strenuous task [19]. Studies in this line of research revealed that higher NFC is weakly to moderately associated with increased Self-Control (*r* ≈ .30 [14,19–21]). However, it was not examined yet, whether this association is valid for different measures and what the processes behind this association are. Previous studies investigating relations of NFC to Self-Control mainly focused on the association itself and its relevance for outcomes like depressive symptoms [14,19–21]. For example, one study examined in which way NFC is related to affective adjustment among 150 university students [14]. In that study, students rated their NFC, dispositional self-control, self-esteem, and habitual depressive mood as indicators of affective adjustment. NFC was found to predict self-esteem and depressive mood indirectly through Self-Control [14]. As in that study, most research used only one self-report measure of Trait Self-Control [14,20,21]. Hence, more research is needed in order to understand the conditions of NFC’s relation to Self-Control by using a broader set of questionnaires and taking into account possible interactions with other variables. Examining such interactions is one way to better understand processes associated with NFC that potentially have implications on successful Self-Control. This approach can provide valuable basic insights in the nature of NFC as well as in processes that contribute to the exertion of Self-Control. One promising link could be the so-called *Action Orientation (AO)*. The dispositional tendency to flexibly recruit control resources, that is AO, has been proximally linked to the exertion of Self-Control (e.g., [22]). Hence, considering AO as disposition related to Self-Control could shed light on the components of Self-Control that are associated with NFC.

The investigation of relations between NFC and Self-Control contributes to research on principles of Self-Control from a personality perspective that so far has only rarely been taken into account [23] as compared to general and social-psychological research (e.g., [3,24,25]). While it is generally accepted that individual differences in Self-Control moderate processes such as the discounting of potential rewards that are delayed or require certain effort (e.g., [26,27]), it is still an unresolved question to what extent Self-Control itself is modulated by dispositional differences in other motivational constructs. As outlined above, by describing differences in cognitive engagement, NFC qualifies as such a construct. In addition, the current study also takes into account another motivational construct, AO, in order to examine how both personality traits contribute to the prediction of Self-Control. In the following, we elaborate on implications of personality traits for Self-Control by providing a general overview of approaches to explain Self-Control and by detailing what is known so far about the relation of NFC and AO to Self-Control.

### Previous research on Self-Control

Self-Control can be defined as process that enables people to restrain from proximal temptations in order to promote long-term goals (e.g., [1]). Sometimes Self-Control and self-regulation are used as interchangeable phrases for the same concept but self-regulation can be also viewed as referring “to the general process by which people adopt and manage various goals and standards for their thoughts, feelings, and behavior, and then ensure that these goals and standards are met” ([1], p. 3). Following this perspective, Self-Control is only one type of self-regulation excluding regulatory processes like regulating motoric actions so that they meet behavioral standards [1]. One popular example of research on Self-Control is the *marshmallow test*. This dilemma between getting a small reward now (e.g., one marshmallow) versus a bigger one later (e.g., two marshmallows) was introduced in the 1960s to examine Self-Control in children and has been used in different variations since then (for overviews, see [28,29]). This test has been questioned to be a pure measure of Self-Control [30] but illustrates a basic characteristic of tasks that demand Self-Control: the conflict between rather global long-term goals or values and short-term situational benefits [1]. Taking this conflict between a current desire and higher-order goals as starting point, several additional steps are necessary to exert Self-Control including control capacity and motivational processes (e.g., [31]). Previous research has shown positive implications of higher Self-Control in real-life settings, for example, regarding physical and psychological health or academic achievement (e.g., [2,28,32]). Given its social relevance, much research in different fields has been carried out to examine the nature of Self-Control and its underlying mechanisms as well as to develop descriptive and explanatory models [1,4,33,34].

Several approaches have focused on distinct cognitive and behavioral processes underlying Self-Control and have outlined a number of complementing mechanisms such as the recognition that a situation requires Self-Control, impulse inhibition, cognitive reconstrual, forming implementation intentions, and conscious attention allocation (for overviews, see [1,35,36]). From a social-psychological perspective, the so-called resource model that proposes a limited resource to underlie Self-Control has been dominating research on Self-Control (e.g., [3]). This model has been criticized for its lacking empirical falsifiability (e.g., [34]) leading to different adaptations and the development of complementary theories (e.g., [4,34]). A rather process-oriented model [37] has emphasized the importance of attentional (e.g., attention on a short-term reward) and motivational processes (e.g., motivation to control), thereby also referring to the awareness of an existing conflict as initial condition for the exertion of control [4]. Other explanations refer to the assumption that Self-Control goes along with perceived intrinsic costs that may be related to the opportunity costs associated with exerting control [4,27,38]. The basic idea of these explanations is that after monitoring a behavioral conflict, successful control depends on whether the subjective value of control outweighs subjective intrinsic costs of the effort needed to control [4].

To sum up, all approaches taken together illustrate that successful Self-Control refers to complex behavioral processes and requires an awareness of the necessity to control as well as the motivational state to not only intend, but to also initiate control behavior. All approaches thus refer to the role of *mental effort* that is needed in order to exert Self-Control (e.g., [3,25]). From a personality perspective, interindividual differences in approaching versus avoiding mental effort are described with NFC as typical investment trait. Hence, NFC has been considered to be relevant for predicting Self-Control in recent research (e.g., [19]).

### Need for Cognition and relations to Self-Control

Referring to the popular resource model of Self-Control [3], it has been argued that the same resource underlies effortful information processing and Self-Control so that engaging in cognitive investments as with higher NFC levels should strengthen this resource and promote Self-Control [19]. However, the resource model has been criticized among others for the empirical vagueness of such a resource and for the impossibility to explain differences in Self-Control and depletion effects that refer for example to motivational processes (for overviews, see [34,39]). Bearing such critical comments on the resource metaphor in mind, this is not the only explanation for a link between NFC and Self-Control. Firstly, NFC has implications for attentional and motivational processes that are addressed in the process-oriented model of Self-Control [37]: Individuals with higher NFC levels tend to process information more comprehensively and elaborately in higher-order cognition (i.e., conscious decision making; [7]) as well as in early attentional processes (e.g., early attention allocation; [8,40]). As individuals high in NFC are assumed to process information in a rather elaborated and less heuristic way [7,8], this should support the detection of behavioral conflicts and recognizing the necessity to exert Self-Control. Secondly, NFC may have implications on motivational processes relevant to Self-Control: Individuals with higher NFC do not only invest more cognitive effort to solve demanding tasks [7] but have been also shown to adapt on higher cognitive demands with increased neural activity [18]. Further, there is evidence that inidivduals value cognitive effort differently depending on their NFC level: In one study, the willingness to expend cognitive effort was measured with a paradigm in which individuals had to choose between larger rewards for cognitively demanding tasks and smaller rewards for tasks that require less cognitive effort [41]. Individuals with higher NFC levels tended less to discount monetary rewards depending on cognitive effort. Hence, this study indicated that higher NFC is associated with evaluating cognitive effort as less intrinsically costly compared to lower NFC [41]. Higher NFC should thereby promote higher motivation to invest the cognitive effort needed to solve behavioral conflicts in the sense of higher-order goals and to resist short-term temptations.

Taken together, the outlined characteristics of different NFC levels in terms of attentional and motivational processes are likely to predict whether people build Self-Control intentions and to what extent they are motivated to act accordingly. However, research on the relation of NFC to Self-Control is still sparse and it is unknown which psychological processes underlie the association of both variables. Whereas the assumed implications of NFC for Self-Control mainly refer to attentional and motivational processes (detecting a need of Self-Control, building intentions to control), the most proximal step in the complex processes preceding Self-Control is the actually invested control effort [31]. Therefore, this study additionally considered AO, which is closely linked to Self-Control research and refers to the effort individuals actually invest to exert control irrespectively of general attentional and motivational processes (for an overview, see [23]). With AO referring to resource recruitment as specific component of Self-Control [31], its interplay with NFC and Self-Control was assumed to substantially contribute to the understanding of processes related to the prediction of Self-Control by NFC.

### Action Orientation

The extent to which individuals recruit control processes is explicitly addressed in the conceptualization of AO. It is defined as an individual’s tendency for the self-motivated initiation and maintenance of intentions related to flexible responses to demanding situations [22,23,42,43]. The opposite pole *State Orientation* refers to a persevering, change-preventing tendency that inhibits to implement intentions, whereas AO as upper pole describes a change-promoting mode and goal implementation that is accompanied by adaptive affective states (e.g., [23]). Thereby, state-oriented individuals have increased difficulties to reduce negative affect and to adaptively adjust control in response to experienced conflicts [22,44]. Three subdimensions of AO are postulated: *Preoccupation* (vs. disengagement) refers to the extent to which individuals are able to disengage from ruminative thoughts or negative affect related to unpleasant experiences (e.g., an experienced conflict), *hesitation* (vs. initiative) describes the general tendency to initiate goal-directed behavior, and *volatility* (vs. persistence) refers rather to maintaining than initiating behavior with high levels enabling individuals to focus on the execution of an intention and to shield intended behavior against distractions [42,44]. Thus, AO is theoretically linked to Self-Control [22,23], which is supported by empirical research on volition [45–47]: Two studies referring to depletion effects after initial Self-Control tasks reported that action-oriented individuals performed better in Self-Control tasks after an initial exertion of control compared to individuals with higher State Orientation [45,46]. Another study showed among 240 young adults that AO moderates the association of executive functions (i.e. cognitive processes enabling goal-directed behavior) with Self-Control [47]. Higher levels of executive functions were stronger related to less real-life Self-Control failures when individuals tended to AO instead of State Orientation. Hence, AO enabled individuals to actually mobilize resources to control [47]. With both dimensions referring to intention initiation, recent research on AO and Self-Control has focused on the failure-related dimension preoccupation (e.g., [44,47]) and on the prospective dimension hesitation (e.g., [45,46,48]).

### The current study

**Fig 1:**
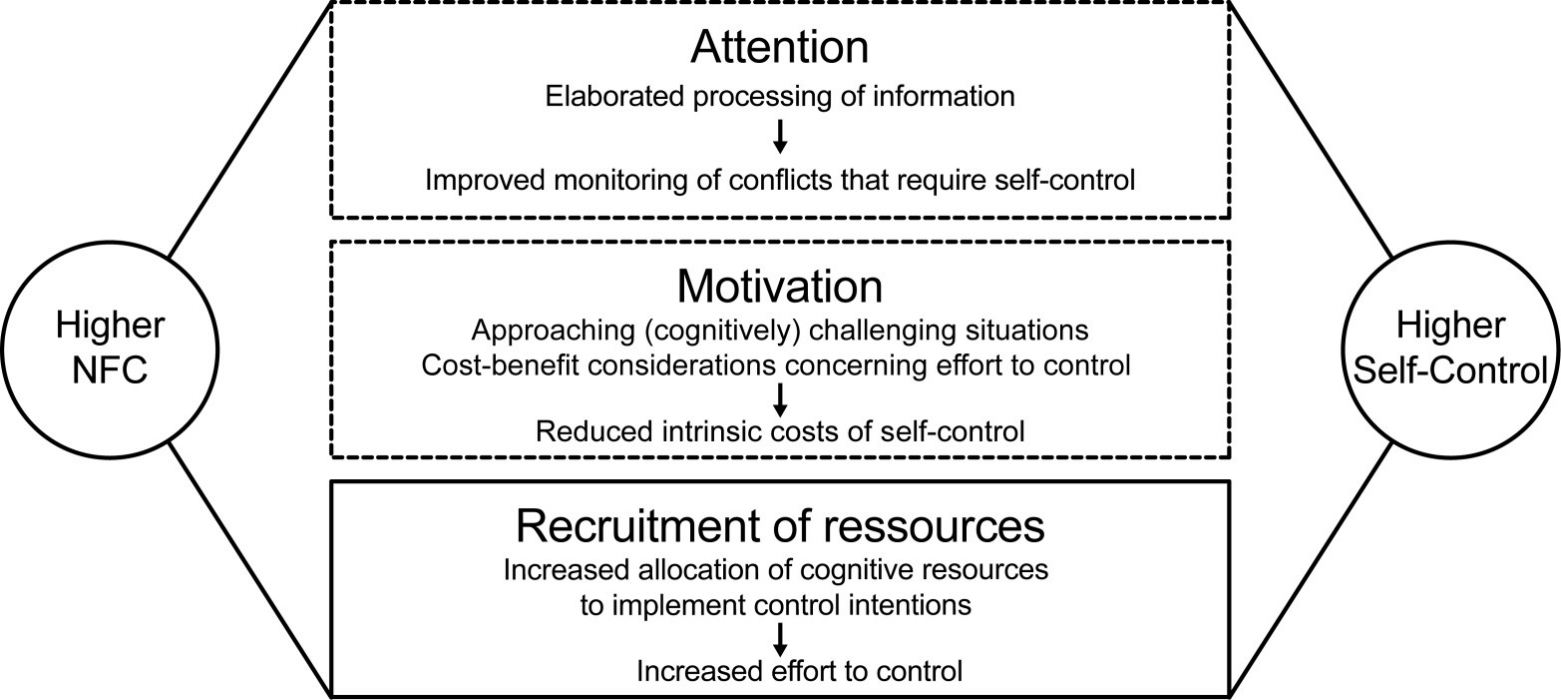
Processes linking Need for Cognition to Self-Control. NFC = Need for Cognition. Illustration of potential explanatory mechanisms that can be derived from literature. The solid-line box represents the role of Action Orientation related to resource allocation for the prediction of Self-Control by Need for Cognition while the dashed-line boxes represent explanatory mechanisms this study did not focus on.

Compared to the large body of research on Self-Control in general, the role of personality for the exertion of Self-Control has been a rather neglected issue. In previous research, NFC (e.g., [14]) and AO (e.g., [42]) were independently related to self-regulatory variables. With the present study, we set out to examine the role of individual differences for the prediction of Self-Control by focusing on NFC in *interplay* with AO. The possible explanations for an association of NFC with Self-Control outlined above are displayed in Fig 1.

Given the theoretical background, one can assume that lower AO would reduce the motivation to invest cognitive effort even in individuals with higher NFC. In turn, higher AO should promote the implementation of control intentions in addition to higher NFC levels. In other words, although higher NFC should be associated with perceiving an existing conflict and with the individual motivation to control, lower AO should reduce the actual control effort and could potentially lead to Self-Control failures despite, for example, sufficient control motivation [31]. In the marshmallow example, higher NFC should promote to become aware of the conflict between two choices and to be motivated to apply a strategy of thinking not about the delicious taste. The level of AO should additionally influence how much cognitive effort an individual would be willing to invest into strategy application: Insufficiently recruited effort could then lead to Self-Control failure in that the individual would not be able to resist the marshmallow’s temptation. Following this thought, AO may moderate the relationship of Self-Control and NFC.

Up to now, there has been no research on the relation of NFC to AO. However, a positive relation between these traits is reasonable when taking into account that NFC is associated with traits referring to goal-oriented behavior [10] and that higher NFC levels go along with cognitive engagement and increased recruitment of cognitive resources [18]. Following this line of reasoning, NFC is related to actual resource recruitment to some extent, which suggests that AO should be positively associated with NFC. That is, by not only approaching cognitively challenging situations but by also increasing cognitive engagement, higher NFC can be assumed to be associated with intensified recruitment of cognitive resources. This assumption automatically links higher NFC to higher AO. Considering that, a (partial) mediation of NFC predicting Self-Control through AO is also possible.

Taken together, we examined the following hypotheses: Based on previous evidence for associations about *r* = .30 of NFC with Self-Control (e.g., [14,19,20]), we expected a positive small to medium association between both constructs. As AO is theoretically related to resource allocation and NFC has been shown to correlate with cognitive effort in demanding situations [18], we expected a positive association between NFC and AO. As engaging in cognitively demanding tasks is only one facet of NFC and the previous study on associations of NFC and cognitive resource allocation reported small to medium effects [18], we expected a small association of NFC with AO. As this relation has not been examined previously, our assumption mainly relied on theoretical reasoning. AO was considered as a potential moderating or mediating variable of the relation between NFC and Self-Control because theoretical considerations provide arguments for both alternatives. Both, evidence for a mediation or a moderation, would foster the understanding of how exactly the personality traits NFC and AO relate to Self-Control and would further contribute to the nomological knowledge about NFC. Hence, we did not favor a specific hypothesis concerning the question whether AO (partly) mediates or moderates the prediction of Self-Control by NFC. As our assumptions were based on theoretical considerations, we employed a confirmatory approach in that we had two concurrent hypotheses for the interplay of NFC and AO (moderation versus mediation).

## Materials and methods

All data and materials for reproducing our primary analyses are permanently and openly accessible at https://doi.org/10.17605/OSF.IO/WN8XM. For documentary purposes of the Technische Universität Dresden, they are also provided via http://dx.doi.org/10.25532/OPARA-19. The hypotheses were not preregistered.

### Sample

A number of 1567 participants began a German online survey, of which 1335 (85%) started to answer the personality measures. Complete data sets were available for 1212 participants. Three participants had to be excluded due to untrustworthy answers: That was, ID 173: gender = „rosa”(German for pink), age = 70, mother tongue = „dänisch”(German for danish; every entry could indeed be true, but their combination seems rather unlikely); ID 503: gender = „Boba Fett”(a headhunter from Star Trek), age = 88 and mother tongue = „dummes Gewäsch”(German for twaddle); and ID 910: age = -13. Hence, the final sample consisted of 1209 participants (58.6% female, 41.1% male; *M*_age_ = 24.43 ± 3.97 years). This clearly exceeded our initial target sample size of *N* = 779 that was determined via G*Power 3.1 [49] to be able to detect even small correlations of *r* = .10 at α = .05 (two-tailed) and 1-β = .80. It resulted in an actual power to detect such small effects of 1- β = .94.

Due to the recruitment procedure (see below), educational level was high with 1198 (99% of *N* = 1209) stating to have a university entrance diploma. Among all participants, 100 Euros were raffled in that two participants could win 25 Euros and one participant could win 50 Euros.

### Procedure

Data were collected with an online survey (LimeSurvey; [50]) that included different personality questionnaires. Participants were invited to take part via a university mailing list. After general study information, all participants created an individual code and answered demographic questions (age, gender, educational level, country of residence in early life, mother tongue). Then, personality variables were assessed. For the current analyses, we focused on the variables NFC, AO, and two measures of Self-Control. We excluded two measures from all analyses that assessed general self-efficacy [51] and the Big Five [52] due to the focus of the current article on examining the specific role of AO for NFC predicting Self-Control.

### Materials

#### Need for Cognition

NFC was assessed using the German 16-item questionnaire [53]. Responses to items (e.g., “Thinking is not my idea of fun”, recoded) were recorded on a 7-point rating scale from -3 (*completely disagree)* to +3 (*completely agree)*. In previous studies, internal consistency was comparably high with Cronbach’s α > .80 [10,53]. Evidence for the scale’s validity comes from, for example, persuasion research where it was shown that the NFC score as measured with the NFC scale significantly interacted with argument quality in that individuals with higher NFC scores more strongly tended to consider the quality of arguments in order to form their attitude [53].

#### Self-Control

We addressed the complexity of Self-Control (e.g., [1,31]) and measurement-related recommendations [54] by considering temperamental Self-Control from a personality perspective that takes into account neuroscientific research [55] as well as Trait Self-Control originated from a broad social-psychological approach [56,57]. Trait Self-Control was assessed with the 13-item version of the German Self-Control scale [56]. It assesses an individual’s perceived capacity for the exertion of effortful control over dominant behavioral responses in the pursuit of long-term goals. Responses to items (e.g., “I am able to work effectively toward long-term goals”) were coded on a 5-point rating scale from -2 (*completely disagree*) to 2 (*completely agree*). The scale shows comparably high reliability (Cronbach’s α ≈ .80, 7-week retest reliability *r*_*tt*_ = .82; [56]). In terms of validity, this scale was positively associated with a measure assessing the more comprehensive construct of self-regulation (*r* = .48 [56]). Referring to external criteria, theoretical expectations were confirmed, for example by medium positive associations with life satisfaction (*r* = .31) and school performance (*r* = .24).

Additionally, the scale Effortful Control from the German Adult Temperament Questionnaire (ATQ) [58] was used. The scale comprises 19 items on executive control in everyday life such as the inhibition of prepotent responses and the resistance to distraction and temptation referring to aspects of inhibitory, attentional, and activation-related control. Responses to items (e.g., “Even when I feel energized, I can usually sit still without much trouble if it’s necessary”) were given on a 7-point rating scale from -3 (*completely disagree*) to +3 (*completely agree*). Internal consistency of the German ATQ has been shown to be acceptable (Cronbach’s α = .74; [58]). Concerning the validation of the German ATQ, Effortful Control was most strongly related to Big-Five Neuroticism (*r* = -.55) and Conscientiousness (*r* = .58), which confirmed theoretically derived assumptions [58].

#### Action Orientation

AO was assessed with a German version of the Action Control Scale [43]. The three dimensions (preoccupation, hesitation, volatility) are assessed with 12 items per dimension. In each item, people are confronted with a situation (e.g., “When I need to solve a difficult problem”) and have to choose the one out of two behaviors that was rather true for them. Responses were scored as state-oriented with 0 (e.g., “I think about other things first before starting with the task at hand”) and as action-oriented with 1 (e.g., “I get started et once”). Research on AO provided evidence for the scale’s validity and reliability including its factor-structure and associations with self-regulation [23,42]. Based on previous research [44,46,47] and because volatility describes tendencies to maintain behavior rather than to initiate implementing (control) intentions (for an overview, see [42]), this study focused on hesitation and preoccupation. For those two subscales, reliability has been good with Cronbach’s α ≥ .70 in previous studies [42,44].

### Statistical analysis

All analyses were done using IBM SPSS Statistics (version 25), RStudio (version 1.1.463), R (version 3.5.2), and MPlus (version 7.11). By using Structural Equation Modeling, we chose a latent variable approach in order to examine relations between NFC and AO to Self-Control that controls for measurement errors and enables conclusions on a construct level [59]. This method was used to compare theoretically derived models with empirical data in order to examine our hypotheses.

#### Parceling procedure

Following the recommendations of Little, Cunningham, Shahar, and Widaman [60], all manifest indicators were parceled and item parcels were constructed by a technique that aims at item-to-construct balance. We used this procedure because this research was not intended to examine the structure of one construct but was interested in relationships at the construct level. Hence, the parceling procedure aimed at optimizing measurement models that represent how parcels of an instrument load on a latent variable captured by each measure (for an overview, [60]). Therefore, separate principal component analyses with a one-factor solution were calculated first for all items of each instrument. Then, items were allocated to different parcels so that all parcels of each instrument had comparable average factor loadings [60]. For AO, the procedure differed slightly from the other instruments because all parcels should load on one latent variable and represent both preoccupation and hesitation to a comparable extent. AO parcels were constructed using a two-step procedure that combined item-to-construct balance and a domain-representative approach [60]. First, factor loadings were calculated separately for both dimensions and ranked. Second and considering factor loadings, items were allocated to four parcels that included three demand-related and three failure-related items each. Parcel values were always calculated as sums. The number of parcels per latent variable was guided by item numbers so that three to four parcels were generated as first-order indicators.

#### Latent interaction modeling

The interaction of NFC and AO was tested using orthogonalized product indicators: First, all possible product terms of predictor indicators (i.e., NFC_parcel_1_ x AO_parcel_1_, NFC_parcel_1_ x AO_parcel_2_, …) were calculated. Second, each product term (e.g., NFC_parcel_1_ x AO_parcel_1_) was regressed on all indicators of NFC and AO. Then, the resulting residuals of these regressions were used as indicators of the latent interaction variable for moderation analysis. Following this procedure, the latent predictor variables did not correlate with the latent interaction term. Correlations were specified between residuals that share common variance because they were created by the same first-order effect indicators. Advantages of this approach are stable model estimates, the availability of fit measures, and the robustness in terms of statistical assumptions (for more details, see [61]).

#### Model estimation

To report and evaluate all estimated models, empirically based recommendations were considered [62,63]. Therefore, multiple indices were used including both incremental fit indices that compare research models with a baseline model assuming independence of all variables and residual-based indices that evaluate the amount of error of model estimation [59,63]. Taking into account previous recommendations and empirical findings about preferred models [59,62,63], a model was considered to have an acceptable fit with Root-mean-square error of approximation (RMSEA) < .07, Standardized Root-mean-square residual (SRMR) < .10, Comparative fit index (CFI) > .93, and Tucker-Lewis index (TLI) > .90. All models were estimated based on the covariance matrix; easier interpretable correlations between all indicator variables are displayed in Table A in S1 Appendix.

### Ethics statement

This study was carried out in accordance with the Declaration of Helsinki. All data were collected during a study on the interrelation of traits related to NFC and Self-Control that was originally planned as preliminary study. That study was initially approved by the local data security administrator. After data collection, an ethics approval was obtained for an essentially similar questionnaire set for a follow-up study (Ethics Committee of the Technische Universität Dresden, EK 3012016) to have an approval for our procedure not only in terms of data security but also in terms of the general procedure.

## Results

### Descriptive statistics and reliabilities

Descriptive statistics of all instruments and parcels are provided in Tables B and C in S1 Appendix. In the current study, reliabilities were estimated with McDonald’s ω for all questionnaires. McDonald’s ω for the overall NFC factor was .86. Reliabilities of Self-Control measures were good with McDonald’s ω = .80 for Effortful Control and McDonald’s ω = .84 for Trait Self-Control. McDonald’s ω for AO was .84. All coefficients indicated comparably good to high reliability.

Mardia’s multivariate skewness was 5.79, *p* < .001, and multivariate kurtosis was 267.98, *p* < .001. Therefore, models were estimated using maximum likelihood estimation with robust standard errors. For analyzing latent interactions, mean- and variance-adjusted maximum likelihood (MLMV) was used. Nested model comparisons were calculated with scaled χ^2^ difference tests.

### Preliminary analyses

**Fig 2:**
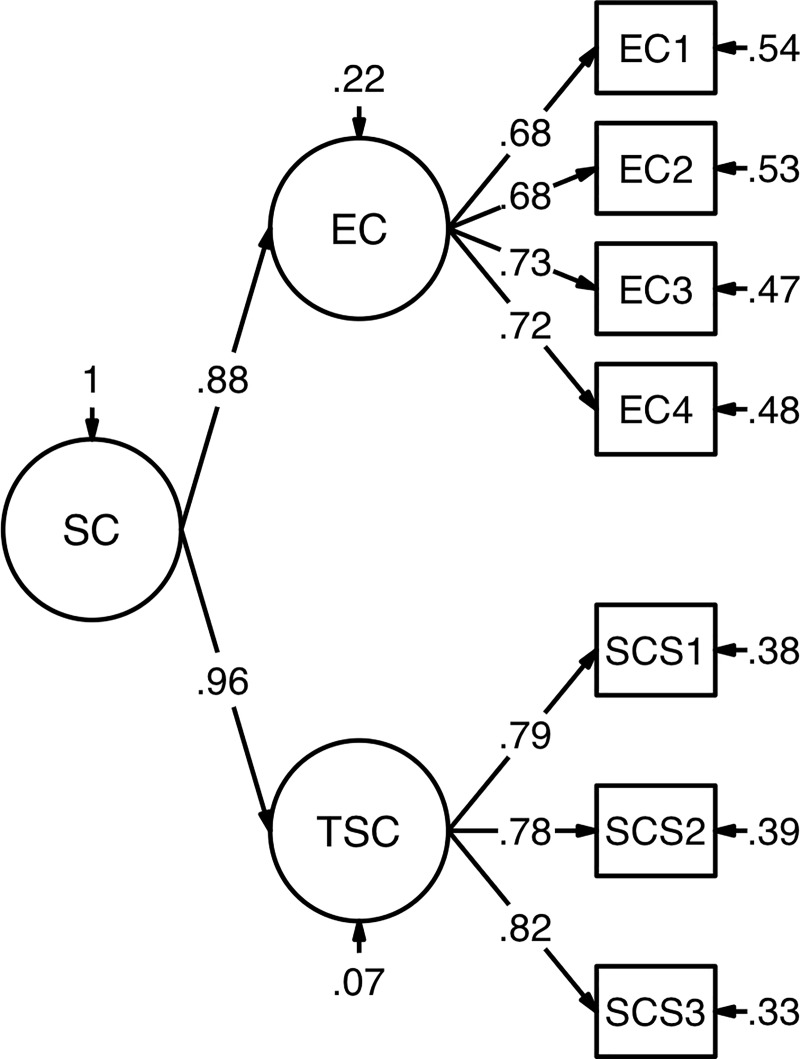
Summary of a second-order factor model of Self-Control. *N* = 1209. Standardized estimates (estimator = robust maximum likelihood), item parcels as manifest indicator variables (see text for details). Unstandardized loadings of first-order factors were set equal. SC = general Self-Control. EC = Effortful Control. TSC = Trait Self-Control. All paths significant with *p* < .001.

Parcels of NFC, Effortful Control, and Trait Self-Control consisted of four or five items; AO parcels included six items each. To verify the quality of the parceling method, average factor loadings of the items on the respective factor were calculated per parcel. The average loadings were at least .50 except for Effortful Control (average loading of parcels between .44 and .48). Then, we determined the measurement model of Self-Control. In this, we tested two models. Following our research interest to examine general Self-Control, the first parsimonious model assumed that all indicators of both instruments to assess Self-Control loaded on one common factor. In comparison, a second-order model took into account that while both instruments focus on different behavioral aspects of Self-Control [56,58], they nevertheless assess a common core construct. It assumed two first-order factors corresponding to both instruments and one second-order factor reflecting the shared variance due to a common Self-Control factor (see Fig 2). This model was more complex, but theoretically favored because it postulates a general Self-Control factor while also representing a substructure that refers to different research traditions and corresponding differences in the specific Self-Control behavior assessed. The hierarchical factor model was chosen instead of a correlated factor model because we were interested in relations of NFC to dispositional Self-Control that should be represented by the common variance of both Self-Control questionnaires. For the second-order factor model, loadings of Effortful Control and Trait Self-Control were set equal due to a high latent correlation of the first-order factors of .85 when replacing the second-order factor by a latent correlation.

Results of the analyses are displayed in Table 1. Indexed by χ^2^-tests (*p* < .001), both models did not fit the data well. As due to the large sample size the χ^2-^test was very likely to be significant, additional indices were taken into account [59]. Cut-offs for RMSEA, SRMR, CFI, and TLI [62,63] were all met for the second-order-factor model, but not for the more parsimonious one-factor model. The superiority of the second-order-factor model was demonstrated by a significant χ^2^ difference test, Δχ^2^_scaled_ = 120.969, Δ*df* = 1, *p* < .001. Thus, the second-order-factor model was used for all subsequent analyses.

**Table 1:** Fit statistics for measurement models of Self-Control.

Model	χ^2^	*df*	*p*	RMSEA [90% CI]	SRMR	CFI	TLI
One-factor model	198.894	14	< .001	.105 [.092, .118]**	.037	.942	.913
Second-order model^a^	69.817	13	< .001	.060 [.047, .074]^ns^	.020	.982	.971

*N* = 1209. All models estimated with maximum likelihood estimation with robust standard errors. Δχ^2^_scaled_ = 120.969, Δ*df* = 1, *p* < .001.

^a^ unstandardized loadings of the first-order factors on general Self-Control were set equal.

** *p* (RMSEA ≤ .05) < .01.

^ns^ *p* (RMSEA ≤ .05) = .104.

### Predicting Self-Control with NFC and AO

**Fig 3:**
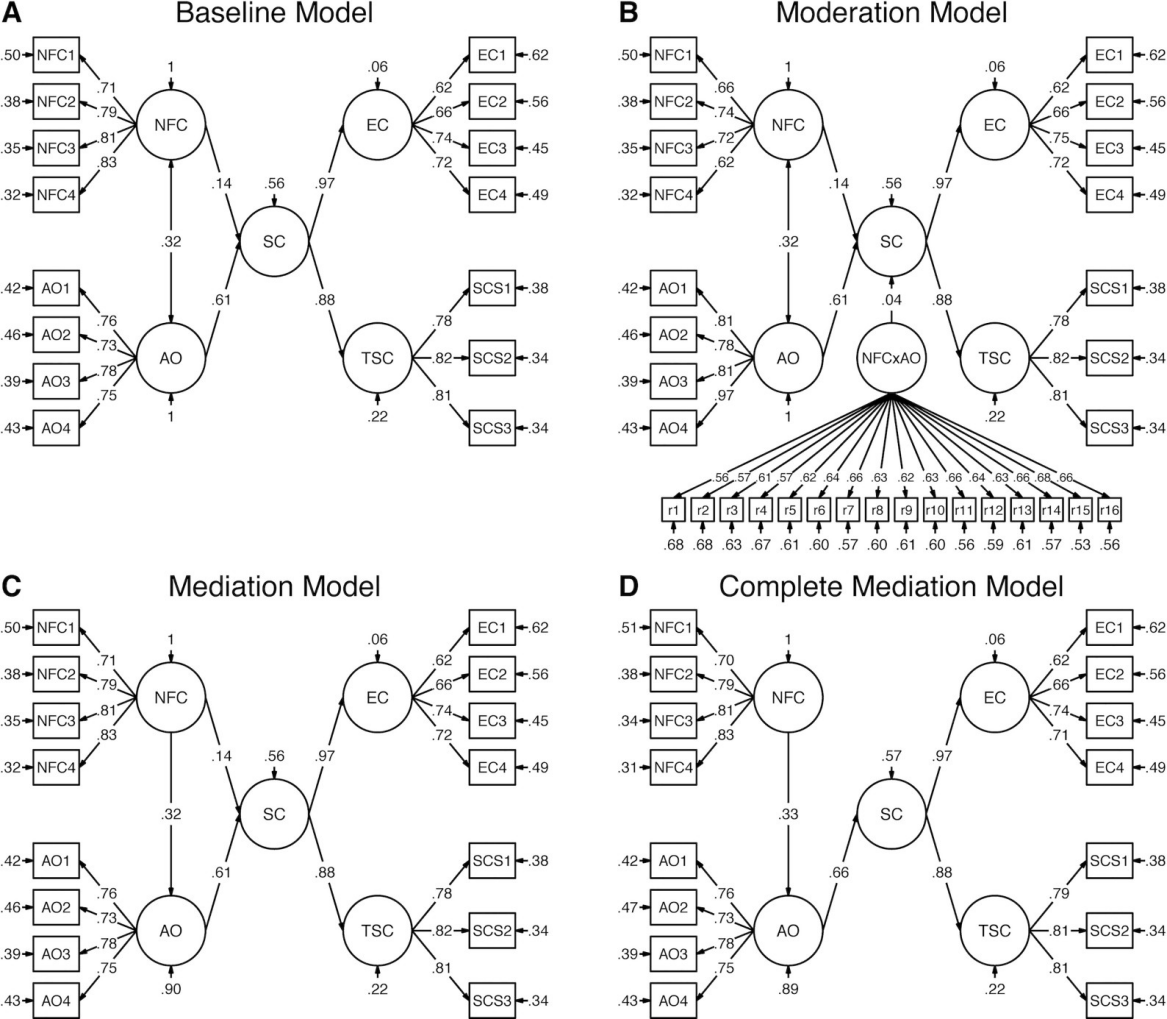
Structural equation models of Need for Cognition and Action Orientation predicting Self-Control. *N* = 1209. Standardized estimates, item parcels as manifest indicator variables (see text for details). NFC = Need for Cognition. AO = Action Orientation. SC = general Self-Control. EC = Effortful Control. TSC = Trait Self-Control. (A) Baseline model. (B) Moderation model, interaction term calculated with residual indicators following the procedure by Little et al. [61], intercorrelations of residuals are not displayed. (C) Mediation Model. (D) Complete Mediation Model. Baseline model (A) similar to mediation model (C) except for the association of NFC with AO being bidirectional in (A) and unidirectional in (C).

First, we tested a baseline model with NFC and AO as correlated predictors of Self-Control that is displayed in Fig 3A. This model showed an acceptable fit with χ^2^ = 465.915 (*df* = 86, *p* < .001), RMSEA = .060 ([.055; .066], *p* = .001), CFI = .953, TLI = .942, SRMR = .050. General Self-Control was predicted by NFC with β = .14 (*p* < .001) and by AO with β = .61 (*p* < .001). NFC and AO were correlated with *r* = .32 (*p* < .001).

In a next step, we tested moderation and mediation models (summarized in Table 2). To test the moderation hypothesis, we followed the procedure by Little et al. [61] and added a latent interaction of NFC and AO to the baseline model. With an excellent fit, this model revealed no significant interaction of NFC with AO (β = .04, *p* = .228), suggesting no moderating effect of AO on the relation of NFC with Self-Control. Mediation was tested by transferring the correlation of NFC to AO into a regression of AO on NFC. The total effect of NFC was β = .33 with the direct effect of NFC (β_dir_ = .14) being comparable to the indirect effect through AO (β_ind_ = .19; all *p* < .001). This model was compared to a more parsimonious model of complete mediation with fixing the direct path of NFC to Self-Control at 0. Given the significant χ^2^ difference test, Δχ^2^_scaled_ = 11.73, Δ*df* = 1, *p* < .001, the model with the direct effect in addition to the indirect path through AO was the superior one. Thus, the model assuming that the association of NFC with Self-Control was partially mediated by AO fitted the data best. All three models are depicted in Fig 3, panels B-D.

**Table 2:** Fit statistics for mediation and moderation models to predict Self-Control.

Model	χ^2^	*df*	*p*	RMSEA [90% CI]	SRMR	CFI	TLI
Moderation NFC x AO^a^	484.93	381	< .001	.015 [.011, .019]^ns^	.030	.989	.987
Partial Mediation NFC → AO → SC^b^	465.92	86	< .001	.060 [.055, .066]**	.050	.953	.942
Complete mediation NFC → AO → SC^b^	485.58	87	< .001	.062 [.056, .067]**	.059	.950	.940

*N* = 1209. NFC = Need for Cognition; AO = Action Orientation; SC = Self-Control. For mediation models, Δχ^2^_scaled_ = 11.73, Δ*df* = 1, *p* < .001.

^a^ estimator = mean- and variance-adjusted maximum likelihood (MLMV).

^b^ estimator = maximum likelihood estimation with robust standard errors (MLR).

** *p* (RMSEA ≤ .05) < .01.

^ns^ *p* (RMSEA ≤ .05) = 1.0.

#### Power and robustness checks

Finally, we addressed the question of the actual power and robustness of the estimates obtained in the partial mediation model. With regard to power considerations, we used the *semTools* package for R to determine the actual power to detect the indirect effect of the partial mediation model following a method described by Satorra and Saris [64] where a model with the observed parameters is compared to a model with the parameter in question—here, the indirect effect—being fixed to zero. Moreover, using a method described by MacCallum and colleagues [65], we calculated the power to achieve our model fit of RMSEA = .06 as compared to an alternative, worse fitting model with RMSEA = .08, and a nearly perfectly fitting model with RMSEA = .01. In all cases, a power of 1 was achieved given our sample size.

**Fig 4:**
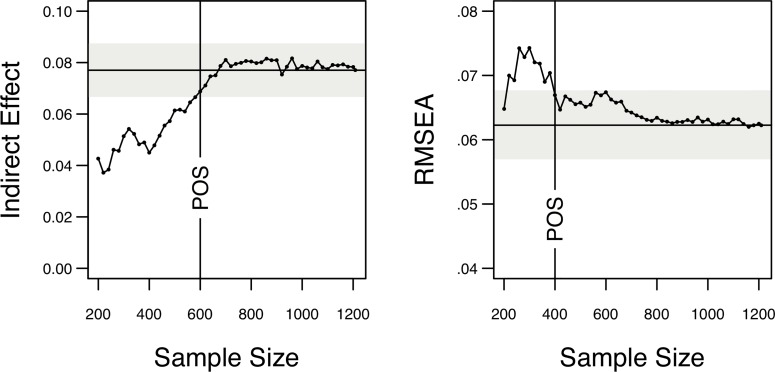
Evolution of coefficients with increasing sample size. Horizontal line = final effect in the total sample. Grey area = corridor of stability. POS = point of stability, i.e., sample size after which the indirect effect did not leave the corridor of stability anymore. Models were fit with sample size starting at *n* = 200 and then increasing by *n* = 20 until the final sample size of *N* = 1209 was reached. Left panel: Evolution of the indirect effect of Need for Cognition on Self-Control via Action Orientation in the partial mediation model, where the corridor of stability is the final effect together ± its final standard error. Right panel: Evolution of the Root Mean Error of Approximation (RMSEA) as fit measure, where the corridor of stability is the 90% confidence interval of RMSEA.

Finally, as robustness check, we applied a procedure inspired by Schönbrodt and Perugini [66] to the partial mediation model and, starting with a sample size of *n* = 200, successively increased the sample size by *n* = 20 until the final sample size of *N* = 1209 was reached. For each sample size, the partial mediation model was fitted to the data and the estimate of the indirect effect as well as the RMSEA was determined. Fig 4 gives the results. As can be seen, the estimate of indirect effect did not leave the corridor of stability as given by the final estimate of the indirect effect ± its standard error anymore at *n* ≥ 600, that is, an estimate of the indirect effect close to the finally found was already obtained with about half of the final sample size. Likewise, the RMSEA did not leave the corridor of stability as given by the 90% CI of the final RMSEA anymore at *n* ≥ 400, meaning that a model fit close to the final fit was achieved already at about one third of the final sample size.

## Discussion

This study examined the role of individual differences in NFC and AO for the prediction of Self-Control. It aimed at replicating previous results of positive associations between NFC and Self-Control (e.g., [14]) and focused on the interplay of NFC and AO. An integrative model of Self-Control identified different components and processes that precede successful Self-Control including, among others, the conflict between a current desire and higher-order goals as starting point as well as the motivation to control desire [31]. Following this model of Self-Control components [31] and the theoretical conceptualizations of NFC and AO, AO was assumed as intervening variable (moderating or mediating). We tested both, a moderation hypothesis and a mediation hypothesis, for the prediction of Self-Control by NFC and AO. In the moderation model to predict Self-Control, NFC and AO did not interact. Instead, we found a (partial) mediation of NFC predicting Self-Control through AO. NFC was moderately related to Self-Control with β = .33 when summing up its direct and indirect effects. The direct effect was β = .14.

### Structure of Self-Control measures

Self-Control was assessed with two questionnaires that refer to Trait Self-Control but comprise slightly different aspects of Self-Control [56,58]. Therefore, we had to test a measurement model of Self-Control first. Both Self-Control measures correspond in that they measure dispositional Self-Control [56,58]. However, they are based on different theoretical backgrounds and hence focus on slightly different behaviors related to Self-Control [56,58]. Following this assumption of shared variance on a higher level and differences on a lower level, we assumed a common (Trait) Self-Control factor divided in two facets: a temperamental facet referring to Effortful Control with relations to basic attentional and cognitive processes [55] and a facet of Self-Control as dispositional tendency in everyday life [57]. Confirming this assumption, our results provide evidence for a hierarchical structure of two first-order factors that indicate both Self-Control facets and one higher-order factor of Self-Control that represents the large amount of shared variance. For the two questionnaires used in our study, this finding confirms previous research on the convergence of different Self-Control measures (e.g., [54,67]). That research [54,67] has outlined that self-report measures of Self-Control share a large amount of variance attributed to dispositional tendencies toward Self-Control whereas performance tasks aim at specific control processes.

### The prediction of Self-Control

To examine how NFC and AO relate to Self-Control, different models were tested referring to Self-Control at the construct level without distinguishing between Self-Control facets. In a baseline model with NFC and AO as coequal predictors of Self-Control, NFC predicted Self-Control additionally to AO (β = .14) but to a lesser degree than AO (β = .61). The moderation model showed no significant interaction, indicating that the prediction of Self-Control by NFC was stable across different AO levels and rejecting the moderation hypothesis. In contrast, there was a mediated effect through AO in that NFC predicted Self-Control with β = .33 in total and had comparable direct and indirect effects (β = .14/.19). The total effect of NFC replicated previous results showing medium associations around *r* = .30 of NFC with Self-Control [14,20–21]. The partial mediation of this association through AO indicates that both traits are relevant for the prediction of Self-Control. Albeit the remaining direct effect of NFC being small, it is remarkable when keeping in mind that AO refers to processes very proximal to exerting Self-Control [31,42,43]. Hence, the direct predictive value of NFC was as small as expected but it still existed when accounting for the final recruitment of control resources represented by AO. This finding indicates that Self-Control depends not only on dispositions that refer to behavior very close to control processes (AO) but also on dispositions that are more broadly related to cognitive processes, such as NFC. On the other hand, it contributes to the understanding of processes that are associated with NFC (and Self-Control) and could explain relations of NFC to Self-Control. Considering key components of Self-Control behavior [31], the current results suggest that different NFC levels have implications on resource allocation and on the engagement not only in general cognition but also in the implementation of control intentions. Moreover, the remaining direct effect indicates that this is not the only process by which NFC and Self-Control are related and argues indirectly for attentional and/or motivational processes related to NFC that predict Self-Control.

### Implications

The present results support the assumption that considering interindividual differences in personality adds to the understanding of the nature and preconditions of Self-Control [23]. The Self-Control measures used in this study refer to observable behavior that indicates (successful) dispositional Self-Control, which can be considered to be a proximate consequence of actually invested control effort. Hence, the higher predictive value of AO compared to NFC fits the conceptualization of AO that is theoretically rooted in research on action control [22,23]. From this perspective, it is even more remarkable that the current results provide evidence for NFC predicting Self-Control in part but not only through AO. The effects in the mediation model underline that both NFC *and* AO are meaningful predictors of Self-Control. Both dispositions are–at least in part–associated with different psychological processes. Hence, the partial mediation can be seen as indirect evidence for different psychological components and processes contributing to how individuals manage behavioral conflicts that demand Self-Control [31].

It indicates that the underlying mechanisms for the relation of NFC to Self-Control do not only refer to an increased tendency to recruit resources to control. Instead, our results provide indirect evidence for relations of NFC with additional attentional and motivational processes relevant to Self-Control (e.g., [31,37]) that are independent from AO and have been addressed in previous research on cognitive correlates of NFC (see Fig 1; [8,18,40,41]). Referring to the marshmallow test and considering different processes that contribute to observable Self-Control [31], NFC may influence (1) to what extent a person is aware of the conflict between one marshmallow versus two, (2) to what extent one is motivated to solve the conflict with increased cognitive engagement, and (3) to whether one actually recruits control resources to manage waiting for the experimenter with strategies like cognitively focusing on the color of a marshmallow instead of the delicious taste.

For personality research in particular, our results suggest an association of higher NFC levels with increased tendencies to AO and thereby with rather flexible responses to situations that demand control. The association between NFC and AO fits with recent results of an increased recruitment of cognitive resources in individuals with high NFC [18]. Thereby, linking NFC to AO and to actually invested effort has implications for research on the nomological network of NFC. It suggests that the engagement aspect of NFC is important not only for thinking in general but also for dealing with situations that demand persistence, focusing on long-term goals, and Self-Control. This finding is additional evidence for an association of NFC and the way individuals use their resources. The conceptual link of AO to affect regulation (for an overview, see [23]) further supports the conclusion of previous research that interindividual differences in NFC can have implications for affective adjustment (e.g., [14,16,17]).

### Limitations and future research

To measure Self-Control, we used two questionnaires that assessed self-perceived Self-Control generalized across different situations. This procedure took into account different theoretical approaches and referred to different behavioral aspects related to Self-Control. However, one could criticize that two self-report measures were used instead of laboratory performance tasks. One pragmatic argument for using self-reports is that they are less time-consuming when researchers aims at large sample sizes as basis for firm conclusions. In contrast to performance tasks, they are likely to provide information representative for real-life behavior across different situations (for a review on measurement characteristics, see [68]). Underscoring this notion and referring to the validity of Self-Control measures, questionnaires should be favored over single performance tasks when facing time or budget constraints [54]. Additionally, the current choice of Self-Control questionnaires was based on our aim to assess Trait Self-Control [54,67]. Following the complementary advantages of self-reports and performance tasks, the usage of questionnaires in the current study facilitated a large sample size and thereby well-founded results. On this basis, future research should examine the generalizability of our results to behavioral assessments of Self-Control.

We examined the interplay of NFC with AO and how both predict Self-Control. The underlying temporal assumption mainly relied on theoretical reasoning and past research whereas the cross-sectional design does not allow for definite conclusions on causality. For NFC, it is quite reasonable to assume a reciprocal relationship to Self-Control. Previous research often assumed NFC as predictor [14,19,20] but there is also initial evidence for reciprocal relations [21]. From a developmental perspective, early Self-Control abilities may influence the persistence of children on cognitive tasks and thereby support higher NFC. For example, early success in Self-Control tasks can increase the subjective opportunities of control and reinforce to exert control effort later on. This subjective view of increased opportunities or less costs of cognitive effort is associated with NFC [41] and may be one mechanism how better Self-Control can promote higher NFC levels. Hence, the idea of reciprocal relations between NFC and Self-Control needs to be tested in the future with longitudinal assessments at different ages.

Following up on the current study, prospective research should continue to include personality traits as predictors for Self-Control and to further elaborate their interaction with situational and conflict-inherent variables (see also [31]). In respect of theoretical explanations for NFC’s relation to Self-Control, future studies have to examine whether attentional and motivational processes associated with NFC levels contribute in the way we assume here. The examination of implemented strategies that foster Self-Control could be an important approach to better understand what conditions lead to successful Self-Control.

## Conclusions

The current study provides insights into how NFC and AO relate to Self-Control and motivates further research on the relevance of interindividual differences for Self-Control. Our results reveal that NFC predicts Self-Control in addition to and partially mediated via AO and highlight the relevance of both AO and NFC for Self-Control research. Remarkably, while AO is conceptualized in the field of volitional research, the origins of NFC as psychological concept are not directly related to research on Self-Control. Together with earlier evidence on the role of NFC in effort investment and Self-Control, the present results broaden the theoretical scope of NFC: They provide evidence that it is associated not only with central information processing and the tendency to approach cognitive challenges but also with the actual investment of cognitive effort in order to successfully implement intentions to exert Self-Control. The relation to effective resource allocation refers to processes that may further contribute to the understanding of how NFC is related to emotional adjustment. The finding of increased effort to control with higher NFC levels is in line with previous research that suggested active behavior to cope with demanding situations as explanation for associations between NFC and subjective well-being (e.g., [15]). From an application-oriented perspective, individuals with higher NFC levels may be better able to implement intentions to adjust on emotional challenges in their everyday life. In the field of therapeutic and counselling issues this can mean that people with lower NFC levels need more support regarding the realization of advices. Prospective studies need to continue our process-oriented approach in order to specify psychological mechanisms associated with NFC that may turn out to be relevant for the coping with cognitive and emotional challenges of individuals.

## Supporting information

S1 Appendix Manifest intercorrelations and descriptive statistics. (DOCX)
