# Supplementary material for: Thinking in action: Need for Cognition predicts Self-Control together with Action Orientation
Source: PLoS One. 2019 Aug 1;14(8):e0220282. doi: 10.1371/journal.pone.0220282 (PMC6675071; doi:10.1371/journal.pone.0220282)
Supplement: S1 Appendix — (DOCX) [file pone.0220282.s001.docx]

|  | 1 | 2 | 3 | 4 | 5 | 6 | 7 | 8 | 9 | 10 | 11 | 12 | 13 | 14 |
| --- | --- | --- | --- | --- | --- | --- | --- | --- | --- | --- | --- | --- | --- | --- |
| 1. NFC P1 | - |  |  |  |  |  |  |  |  |  |  |  |  |  |
| 1. NFC P2 | .59 | - |  |  |  |  |  |  |  |  |  |  |  |  |
| 1. NFC P3 | .54 | .63 | - |  |  |  |  |  |  |  |  |  |  |  |
| 1. NFC P4 | .57 | .64 | .70 | - |  |  |  |  |  |  |  |  |  |  |
| 1. AO P1 | .26 | .20 | .19 | .19 | - |  |  |  |  |  |  |  |  |  |
| 1. AO P2 | .19 | .11 | .15 | .13 | .54 | - |  |  |  |  |  |  |  |  |
| 1. AO P3 | .22 | .17 | .20 | .19 | .59 | .61 | - |  |  |  |  |  |  |  |
| 1. AO P4 | .26 | .19 | .21 | .21 | .60 | .55 | .57 | - |  |  |  |  |  |  |
| 1. EC P1 | .25 | .17 | .16 | .14 | .41 | .40 | .40 | .42 | - |  |  |  |  |  |
| 1. EC P2 | .17 | .18 | .16 | .11 | .26 | .26 | .28 | .25 | .46 | - |  |  |  |  |
| 1. EC P3 | .28 | .21 | .15 | .17 | .46 | .41 | .42 | .39 | .50 | .50 | - |  |  |  |
| 1. EC P4 | .25 | .23 | .19 | .19 | .34 | .31 | .33 | .35 | .50 | .51 | .51 | - |  |  |
| 1. TSC P1 | .21 | .12 | .07 | .06 | .32 | .30 | .35 | .32 | .43 | .44 | .46 | .51 | - |  |
| 1. TSC P2 | .34 | .27 | .20 | .16 | .35 | .29 | .33 | .33 | .48 | .45 | .54 | .51 | .59 | - |
| 1. TSC P3 | .32 | .19 | .16 | .16 | .32 | .27 | .32 | .26 | .46 | .49 | .50 | .44 | .67 | .63 |

**Table A. Intercorrelations of Manifest Indicator Variables.**

*N* = 1209. All correlations significant with *p* < .001. NFC= Need for Cognition; AO = Action Orientation; EC = Effortful Control; TSC = Trait Self-Control; P = parcel.

**Table B. Descriptive Statistics of Personality Variables.**

|  | Need for Cognition | Action Orientation^a^ | Effortful Control | Trait Self-Control |
| --- | --- | --- | --- | --- |
| *M* | 16.62 | 9.84 | 6.84 | 0.12 |
| *SD* | 13.19 | 4.96 | 13.97 | 8.36 |
| Skewness | -0.65 | 0.33 | -0.08 | 0.06 |
| Kurtosis | 0.76 | -0.39 | 0.28 | -0.25 |
| Range | -43 – 48 | 0 – 24 | -45 – 57 | -22 – 24 |

*N* = 1209. All scores calculated as item sums. Standard errors for skew and kurtosis are 0.07, and 0.14, respectively.

^a^ score of all items referring to the failure-related and prospective dimensions of Action Orientation.

**Table C. Descriptive Statistics of Parcels.**

|  | *M* | *SD* | Skewness | Kurtosis | Range |
| --- | --- | --- | --- | --- | --- |
| NFC P1 | 3.95 | 4.07 | -0.56 | 0.23 | -12 – 12 |
| NFC P2 | 4.46 | 3.97 | -0.55 | 0.24 | -11 – 12 |
| NFC P3 | 4.98 | 3.62 | -0.78 | 1.06 | -12 – 12 |
| NFC P4 | 3.24 | 4.02 | -0.36 | 0.00 | -12 – 12 |
| AO P1 | 2.24 | 1.51 | 0.40 | -0.45 | 0 – 6 |
| AO P2 | 2.38 | 1.46 | 0.37 | -0.46 | 0 – 6 |
| AO P3 | 2.37 | 1.51 | 0.35 | -0.51 | 0 – 6 |
| AO P4 | 2.85 | 1.53 | 0.09 | -0.69 | 0 – 6 |
| EC P1 | 1.05 | 3.99 | -0.14 | 0.02 | -11 – 12 |
| EC P2 | 1.37 | 4.53 | -0.17 | 0.15 | -13 – 15 |
| EC P3 | 1.02 | 4.47 | 0.06 | -0.11 | -12 – 15 |
| EC P4 | 3.40 | 4.74 | -0.23 | -0.04 | -14 – 15 |
| TSC P1 | -0.17 | 3.16 | 0.11 | -0.47 | -8 – 8 |
| TSC P2 | 0.91 | 3.00 | -0.14 | -0.33 | -7 – 8 |
| TSC P3 | -0.62 | 3.46 | 0.07 | -0.30 | -10 – 10 |

*N* = 1209. All parcels calculated as item sums of four to six items. Standard errors for skew and kurtosis are 0.07, and 0.14, respectively. NFC = Need for Cognition; AO = Action Orientation; EC = Effortful Control; TSC = Trait Self-Control; P = parcel.

^a^ parcels referring to the failure-related and prospective dimensions of Action Orientation.
